# Understanding the feelings and experiences of patients with periodontal disease: a qualitative meta-synthesis

**DOI:** 10.1186/s12955-022-02042-5

**Published:** 2022-08-26

**Authors:** Jun Yin, Yan Li, Mingyu Feng, Li Li

**Affiliations:** Nanjing Stomatological Hospital, Medical School of Nanjing University, Nanjing, 210008 Jiangsu China

**Keywords:** Experiences, Treatment, Periodontitis, Qualitative, Systematic review

## Abstract

**Background:**

Patients’ experiences, feelings, and perceptions play key roles in quality of life and dental care quality, but they are poorly understood in periodontal disease. Therefore, this meta-synthesis aimed to gain deep insights into the feelings, experiences, and perceptions of people living with periodontal disease.

**Methods:**

Electronic database searches in PubMed, Cochrane Library, EMBASE, Scopus, Web of Science, PsycINFO, CINAHL, and Open AIRE were conducted up to December 2021 (updated in June 2022). The JBI Critical Appraisal Tool was used for quality assessment. Then reviewers integrated findings from qualitative studies with a thematic synthesis approach.

**Results:**

A total of 567 studies were identified, of which eight involving 131 participants met the inclusion criteria. Studies were conducted between 2008 and 2021within Europe (Sweden and UK), Asia (Korea, Indonesia, and Singapore), and Oceania (New Zealand). Three analytical themes with nine descriptive themes emerged from the qualitative data. The themes were as follows: (1) “pressure (physical, psychosocial, and financial),” (2) “coping and adaptation (avoidance of the status quo, trying to understand it, and taking responsibility for their own),” (3) “reflection and evaluation (exploring the causes, personal control, and calling for better dental care).”

**Conclusions:**

This review provides insights into how patients perceive and cope with periodontal disease. The findings highlighted patient-centered care in PD, and based on the findings, it is possible to provide more precise and efficient interventions for better patient compliance and treatment outcomes.

**Supplementary Information:**

The online version contains supplementary material available at 10.1186/s12955-022-02042-5.

## Background

Periodontal disease (PD), defined as chronic inflammatory conditions that affects the tissues surrounding and supporting the teeth, is one of the world’s most common diseases [1]. Globally, the prevalence of PD is up to 50% [2, 3]. Once people got PD, they are at increased risk of tooth loss, masticatory dysfunction, and other problems, which can negatively impact their nutrition, systemic health, quality of life, aesthetic appearance, and even self-esteem [3–7].

Nowadays, more and more researchers are focused on patient-reported outcomes (PROs), and trying to promote biopsychosocial aspects of patients’ health. Up to now, the most important PROs in dental practice are oral health-related quality of life (OHRQoL) [8]. According to an umbrella review, PD is negatively correlated with OHRQoL, which includes physical, psychological, and social aspects [4]. However, these studies are mainly quantitative studies based on PROs measuring tools. Although these studies can help us understand how patients are affected by PD, when it talks about complex phenomena like their real thoughts, emotions, and attitudes, the findings from quantitative studies may not be enough [9]. In particular, in order to provide quality health care, it’s necessary to incorporate patients’ perceptions into treatment planning and execution [10].

Moreover, researchers in a previous review pointed that to get further insight into the patients’ perception of oral health, different methods should be used [11]. Qualitative methods can provide insight into patients’ lifeworld, including all their mental and physical health conditions [12]. Nowadays, an increasing number of scholars worldwide are focusing on the subjective experiences of patients with PD and their perceptions on the disease and its treatment. A synthesis of such qualitative studies can paint a rich, subtle and useful picture of patients’ experiences, views, or beliefs. Thus, researchers can "go beyond" the individual findings of primary research and generate novel findings greater than the sum of all of them [13, 14].

To the best of our knowledge, there is no published meta-synthesis pertaining to the feelings, experiences, and perceptions of individuals living with PD. However, to design targeted interventions to help patients understand and cope with the disease, dental practitioners should first understand their perceptions and experiences. Hence, we conducted this meta-synthesis to gain in-depth insights into the feelings, experiences, and perceptions of people living with PD.

## Methods

Qualitative meta-synthesis involved the following key steps: (i) Structured research questions, (ii) Rigorous search strategy and screening, (iii) Data extraction and quality appraisal, (iv) Team-based data synthesis. The selection process was summarized in the Preferred Reporting Items for Reviews and Meta-Analyses (PRISMA) flowchart, and the reports followed the Enhancing Transparency in Reporting the Synthesis of Qualitative Research (ENTREQ) guidelines [15]. This review was registered in PROSPERO (CRD42022297629).

### Research question and selection criteria

The research question is: What are the experiences, feelings, and attitudes of people living with PD? We defined the inclusion and exclusion criteria based on the PICoS framework, which stands for the Population, the Phenomena of Interest, the Context and the Types of Study [16].

The inclusion criteria were: (i) The research Population were patients with PD, including periodontitis and gingivitis, (ii) The Phenomena of Interest in this study were the experiences, feelings, attitudes, and views towards diagnosis and treatment, (iii) The Context including home, social settings, or dental clinic departments, and (iv) The Types of Study were published or unpublished qualitative studies.

The exclusion criteria were: (i) Commentaries, books, conference papers, reviews, letters, (ii) Articles involving people with complex systemic problems, such as cancer, intellectual or cognitive impairments, and so on, (iii) Mixed-method studies in which qualitative results could not be analyzed separately from the quantitative results, and (iv) Non-English papers.

### Search strategy

A comprehensive electronic search was conducted on eight databases including PubMed, The Cochrane Library, EMBASE, Scopus, Web of Science, PsycINFO, CINAHL, and Open AIRE. The publication date was limited to the date of inception to December 2021. All the databases were updated in June 2022. References of selected studies were screened manually and hand-searching for additional studies was conducted. The subject index terms are “periodontal disease*”, “gingival disease*, “gingival recession*, “gingivitis, “gingival pocket*, “periodont*, “periodontitis”, “experience*”, “feeling*”, “attitude*”, “perception”, “psycho*”, “qualitative research”, “qualitative study”, and so on. More detailed search strategies can be found in the appendix (Additional file 1: Table S1).

One reviewer (R1) conducted the search based on a priori search strategy. Then two independent reviewers (R1 and R2) screened the titles and abstracts after duplicates were removed through EndNote X9. Next, the two reviewers retrieved and assessed relevant articles after reading full texts. During the whole process, we kept discussing to resolve some disagreements.

### Data extraction

Data were extracted into Microsoft Excel by one reviewer (R1) and verified by two authors (R2 and R3). Disagreements were resolved by discussion among the reviewers (R1, R2, and R3). One reviewer (R1) contacted the primary investigators of the included studies for some missing or unreported data. The summary table included publication information (author’s name, year of publication, and country of publication), methodological characteristics (study design, study setting, data collection and data analysis), characteristics of the sample (sample size, gender, and age), and key findings of the studies.

### Risk of bias (quality) appraisal

The Joanna Briggs Institute (JBI) Critical Appraisal Tool for qualitative research was used was used to ensure transparency and reliability of the study findings. The tool includes ten items (regarding ethics, possible biases brought by the researchers, the integrity of the methodology and congruity between the research objectives, methods, results, and conclusion) with four options (yes, no, unclear, or not applicable) [17]. We counted the answer “yes” in each article to facilitate evaluation and interpretation of the studies’ findings. Two independent reviewers (R1 and R2) did the quality appraisal and discussed some disagreements. However, to obtain depth and richness of data, we included all studies regardless of their methodological quality.

### Strategy for data synthesis

This meta-synthesis followed the thematic synthesis methodology [18]. The three reviewers (R1, R2, and R3) coded the primary text results line by line by repeated reading, and then we discussed the similarities and differences in coding. Next, we organized the free codes into descriptive themes by inductive analysis. At last, we developed analytical themes based on discussion. We focused on patients’ experiences, feelings, and attitudes when synthesizing. To facilitate the report of synthesis, we used tables and figures. After synthesis, we conducted a team-based reflection, which involved individual reflexive journaling and group discussions. We presented examples of the data synthesis process in the appendix (Additional file 1: Table S3).

## Results

### Search results

**Fig. 1:**
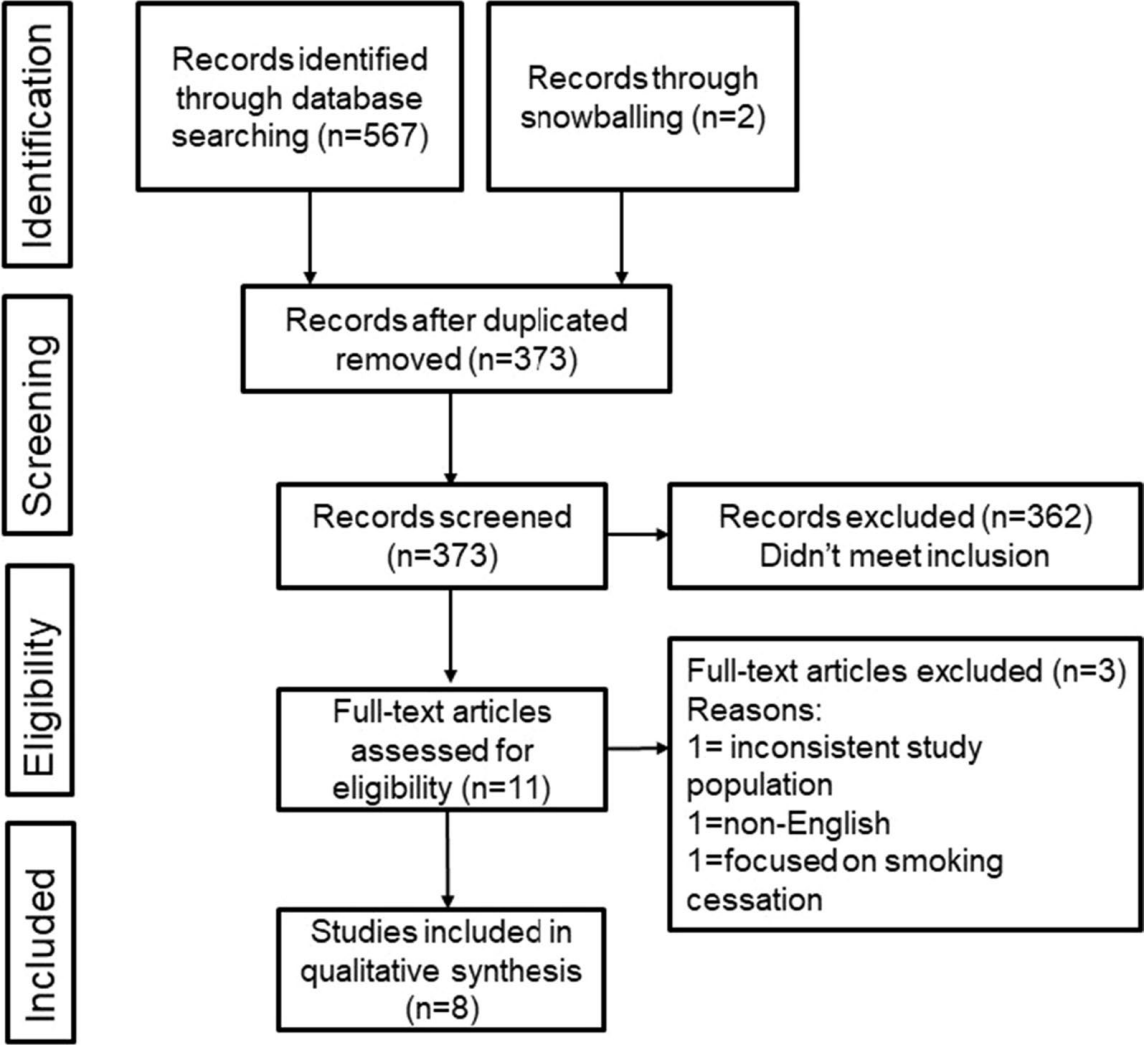
Prisma flow chart depicting the search strategy of the studies included in the meta-synthesis

A total of 567 articles were initially searched and two additional articles were acquired while full-text level screening. Of these, 362 were excluded after reading titles and abstracts. A total of 11 records were assessed by reading full texts. At last, eight articles were included and analyzed for meta-synthesis. The retrieval searching process of the review is shown in Fig. 1.

### Study characteristics and quality appraisal

All of the included studies (Table 1) were qualitative studies. They were conducted in Europe (n = 4; 3 in Sweden and 1 in the UK), Asia (n = 3; 1 in Korea, 1 in Indonesia, and 1 in Singapore), and Oceania (n = 1, New Zealand), published between 2008 and 2021. A total of 131 participants (Men = 56, Women = 75) were comprised in the studies. Interviews were used in all included studies, and dairies were used in one study additionally. Thematic analysis and the constant comparative method were used most frequently (n = 6, 75%). Other studies (n = 2, 25%) did not specify which analytical method was used, but they described the detailed analytical steps.

**Table 1:** Characteristics of the included studies

Study	Country	Study design/setting	Data collection/analysis	Sample size (men, women)/Age	Key findings
Abrahamsson [24]	Sweden	Grounded theory/clinic	Open interview/constant comparative method	17 (7, 10) 42–68y	A core concept: keeping up appearance and self-esteem 1. Doing what you have to do, trying to live up to the norm 2. Suddenly have a shameful and disabling disease 3. Feeling deserted and in the hands of an authority 4. Invest all in a treatment with an unpredictable outcome
Karlsson [25]	Sweden	Phenomeno-graphic approach/clinic and participant’s home	Semi-structured interview/identify & mark, condense, and compare & name	10 (5, 5) 34–78y	1. Perceptions of disease 2. Perceptions of having the disease under control
Stenman [26]	Sweden	Grounded Theory/outside the clinic	Open-ended interview/constant comparative method	16 (7, 9) 41–68y	A core concept: Understanding the seriousness of the disease condition 1. The need to be treated respectfully 2. To gain insight 3. Frustration about the financial cost for treatment 4. Feelings of control over the situation
O'Dowd [19]	UK	Qualitative design/non-clinical setting	Semi-structured interview/constant comparativemethod	14 (6, 8) 29–65y	1. Impairment 2. Functional limitation 3. Discomfort 4. Disability 5. Stigma 6. Retrospective regret
Horne [20]	New Zealand	Qualitative design/conference room	Diary, semi-structured interview/inductive thematic analysis	14 (5, 9) 35–68y	A core theme: progression to a more positive outlook 1. Concealment 2. Having a guilty conscience 3. Patient comfort as paramount
Pyo [21]	Korea	Qualitative design/hospital conference room	In-depth semi-structured interview/data segmentation and then categorization	19 (7, 12)The 40–60s (40–69y)	1. Interfering element for dental care 2. Declined quality of life caused by dental disease 3. Satisfaction elements after treatment of dental disease 4. Improvements for voluntary dental care
Hijryana [22]	Indonesia	Qualitative design/non-clinical setting	Semi-structured interview/thematic analysis	31 (15, 16) 60–80y	1. Impairments related to PD 2. Pain and physical discomfort related to PD 3. Functional limitations and physical activity restriction as a result of PD 4. Psychological discomfort as a result of PD 5. Psychological disability as an impact of PD 6. Social disability due to oral health problems
Wong [23]	Singapore	Qualitative design/office setting of centre for oral health	In-depth semi-structured interview/inductive thematic analysis	10 (4, 6) 22–58y	1. Knowledge of PD and its relationship with 2. Systemic health and QoL 3. Experience and perception on how periodontal treatment can improve QoL 4. Perceived value of having a disease- specific QoL instrument for PD

We only used the JBI Critical Appraisal Tool because there was no mix-method study. The methodological quality of all included articles was scored 7–9, which means at least seven out of ten items were “yes” (Additional file 1: Appendix Table S2). All studies reported in detail the research questions, objectives, data collection, data analysis, interpretation of results, characteristics of the study population, ethical issues, and conclusions. However, five studies failed to state philosophical perspective clearly and only reported the study was qualitative or used qualitative methodology [19–23]. Additionally, all included studies except two [20, 22], failed to discuss the role of the researchers and their influence on the research.

### Meta-synthesis

**Fig. 2:**
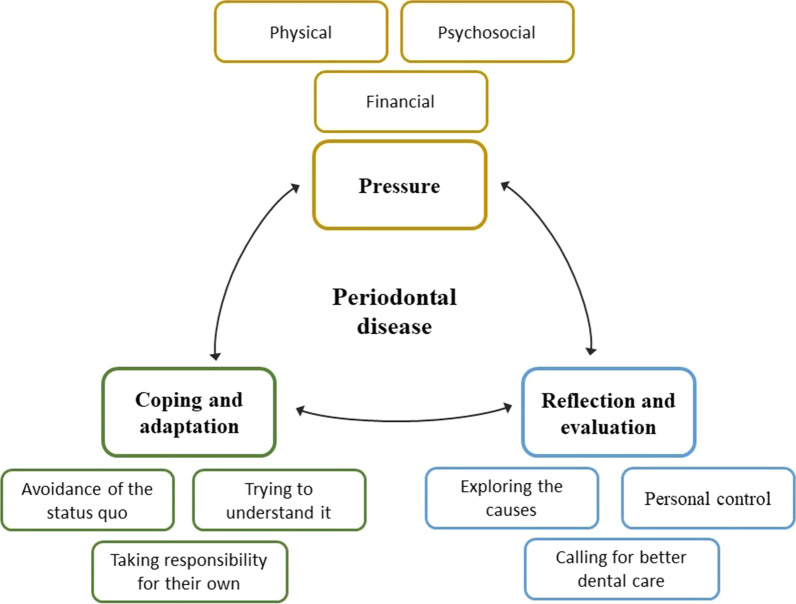
Thematic diagram framework

We identified three analytical and nine descriptive themes from the analysis of eight eligible papers (Fig. 2). The main themes are entitled “pressure,” “coping and adaptation,” and “reflection and evaluation.” For patients with PD, these three themes interact with each other. “Pressure” is caused by the disease and its treatments, “coping and adaptation” is a response to pressure and self-reflection; the process of “reflection and evaluation” is activated from the moment the symptoms appear. Combining all these themes, we can explain the feelings, experiences, and perceptions of patients with PD.

### Theme 1: pressure

This analytical theme depicts the various impacts of PD on patients through three descriptive themes: “physical,” “psychosocial,” and “financial.”

#### Physical

Owing to physiological changes, patients with PD experienced a variety of symptoms, most of which was masticatory discomfort. For example, patients reported inability to masticate, tooth sensitivity, toothache, etc. [19, 20, 22] As a result, some of them had to change their eating habits and avoid masticating which they used to eat, as one patient expressed “I cannot bite into an apple and eat it that way, I have got to chop it up...”[19] (P2) Some patients started masticating unilaterally [22], which may have an impact on their appearances. Moreover, the consequences of periodontitis, such as the destruction of periodontal support tissue and tooth loss subsequently, can further affect the appearance [22]. Some patients were quite concerned about their bad breath and feel embarrassed [19, 22]. Especially when they were in a relationship, halitosis can make them very upset, “The wife mentioned something like ‘Your breath smells’, I feel a bit self-conscious.” [19] (P7).

#### Psychosocial

With these symptoms, patients usually experienced a range of negative feelings. When they were diagnosed with periodontitis, the patients expressed the feeling as “a shock,” even though they had already had the problem for a long time. Then, they perceived that there was too much to fear. Some patients worry about tooth loss, “I was so afraid that my teeth would fall out there,” [22] (P9) and some fear that the situation is a sign of faint, “I didn’t feel dizzy, but I felt I could faint because too much blood came out [from her gums].”[22] (P4) In some cases, patients considered it a stigma. One patient reported, “I’ve always felt there was like a stigma about it [PD].”[19] (P13) Therefore, some reported the disease had affected their self-confidence a lot [22]. When they communicated with others, they were extremely uncomfortable, and some unconsciously covered their mouths [21, 22], Moreover, as some cases reported, PD have limited patients’ daily life in many ways [21, 22, 24, 25], “All of my activities were interrupted, I couldn’t do anything.” [22] (P16).

#### Financial

In studies from Sweden [24, 26] and Korea [21], some patients expressed the financial burden of periodontal treatment. They expressed their frustration with the health insurance system and their sadness over the high costs, “The cost is too high. I thought it was 2.5 million won as a total, but if the pillar (implant fixture) takes 1 million won, then it would cost me another pretty penny for visiting back and forth.” [21] (P1).

### Theme 2: coping and adaptation

This analytical theme is built on the theme of “pressure” and emphasizes the dynamic and balanced process of the disease and its treatment. It can be interpreted by three descriptive themes: “avoidance of the status quo,” “trying to understand it,” and “taking responsibility for their own.”

#### Avoidance of the status quo

Some deliberately avoided discussing their oral conditions because of the perceived stigma [19, 20, 24]. They felt embarrassed to be diagnosed with PD; some would rather say “my bone’s crumbling” rather than “I’ve got gum disease.” [19] (P13) “I’m reluctant to talk about it. Only very close people really know about it,” [20] (P11) a patient stated. On the other hand, facing various embarrassing symptoms such as halitosis, some disguised it by chewing gum [20].

#### Trying to understand it

Others took positive approaches to cope with the disease. They got knowledge from various media (internet, TV, radio, etc.) and learned from their interactions with doctors [23]. During their treatment, some of them tried everything to understand how their disease developed with the presentations of radiographs, photographs, and brochures. “The participants had obtained immediate feedback regarding this matter and, if needed, complementary information and instructions,” as the author stated [26].

#### Taking responsibility for their own

Most patients gradually realized their own responsibility in their struggle against the disease, therefore, they devoted a lot of time, money and energy consciously to regain their health [20, 21, 25, 26]. As one stated, “They have done the hard work and scraping, so it’s up to me now to continue and keep the hygiene side of it up.” [20] (P14) They gradually developed healthy oral hygiene habits, as they reported that they became more careful in brushing their teeth [21] (P10).

### Theme 3: reflection and evaluation

This analytical theme can draw a picture of how patients understand their disease and treatment. This process runs throughout the disease and treatment. Three descriptive themes can help illuminate the process, namely “exploring the causes,” “personal control,” and “calling for better dental care.”

#### Exploring the causes

Their perceived causes of the disease were multiple. Most patients felt that previous unhealthy behavior (e.g. smoking, irregular checkups, etc.) should account for their current condition [19, 20, 24]. Some patients had visited the dentist before, but they felt those doctors failed to inform them of their oral condition adequately and oral health maintenance properly. As a result, their oral condition got worse [21, 24]. Two studies [21, 23] reported subjective obstacles to get dental care; one patient stated that he was fear of anesthesia and thus couldn’t go to the dentist[21] (P6). One study from Korea[21] reported that lack of dental-related knowledge was a cause, which could account for their poor dental care behaviors; one living in the countryside complained that he had no concept of brushing until high school [21] (P3).

#### Personal control

Personal control emphasizes the degree of confidence one has in controlling the progression of the disease, including the perception of whether the disease can be cured or treated [27]. We found that patients’ personal control over the disease varied among stages and patients [20, 21, 23–26]. Some patients knew little about the disease when they first learned of their diagnosis and needed some time to understand it [24, 25]. In their primary opinion, PD was a natural process related to aging. Moreover, they were unaware that PD was the main cause of tooth loss [25]. However, when they learned more about the disease, they gradually realized their own responsibility in oral health, so their sense of control increased to some extent [26]. Furthermore, they found harmonious doctor-patient relationships increased their confidence in controlling the disease [20] (P14).

Their sense of control over the treatment was different. On the one hand, some patients reported they lacked a sense of control over the disease after experiencing treatments from different dentists. Therefore, they felt frustrated and had to rely on authority [24]. Besides, a minority were disappointed when they found the treatment was not as effective as they expected, namely it had just slowed the progression in their view. As one patient stated, “I had hoped that the treatment would help more, but it’s just slowed it down a little.” [26] On the other hand, however, the majority had positive attitudes towards the treatment. They felt the treatment gave them a better sense of control over the disease [21, 23–26]. As one patient stated, “After that (the treatment), my world changed upside down.” [21] (P13).

#### Calling for better dental care

Reflecting on the whole process from the patient's perspective, we can make some improvements. Some patients would like more public education about the disease, so they can give attention to oral health and take action earlier [21]. Some patients stated it was necessary to train professionals in communication to provide effective health education [25, 26]. “They have to learn to hold a serious dialog…they should learn a little more about dealing with people.” [26] They wanted to be treated with respect and encouraged, rather than leaving them in shame and humiliation while communicating with their dentists; one patient expressed that they were already aware that they had made mistakes, so wished there was no more blame [26]. Further, physical and mental pain during the treatment was another thing to consider [20, 25]. One patient stated the feeling, “Stuck in the chair with the lights… the bite block… the noise… the scraping and the pain.” [20] (P9) Some patients perceived the impact of PD on their quality of life and felt it was necessary to develop a disease-specific quality of life instrument [23]. “It helps the dentist know me better,” one patient stated in a recent study [23] (P9).

## Discussion

This paper used a meta-synthesis approach to review eight studies on the feelings and experiences of patients with PD. Based on the repeated reading, analysis, and discussion, we generated three analytical themes, which were entitled “pressure,” “coping and adaptation,” and “reflection and evaluation.” All the themes can give an insight into how PD affects patients and how they understand and cope with it, which can offer implications for better dental care.

Our findings reveal that PD acted as a stressor and usually caused physical and psychosocial impacts on patients. Similar to the findings from quantitative studies based on OHRQoL, these patients experienced poor symptoms, which can affect their function, subjective comfort, and self-confidence [11, 28, 29]. As a systematic review found, however, this condition can be improved significantly and remain stable in the short term when patients get non-surgical periodontal treatment [30]. Our findings from qualitative studies are not limited to the quality of life. To our knowledge, some psychosocial aspects have not been studied in this group.

Uniquely, some patients felt the disease stigmatized them because only those in poverty would get it [19, 20, 24]. In that case, patients’ perceptions of the disease resulted in the negative experience. Similarly, Bitencourt et al. found that people’s perceptions of tooth loss can determine how much they were affected by this experience [31]. Therefore, it can be practical for professionals to get patients’ perceptions of the disease, which can help reduce these psychosocial impacts.

Other pressure on the patients came from the financial aspect. Some people from Sweden and Korea expressed heavy burdens because of the periodontal treatment [21, 24, 26]. To our knowledge, this was rarely reported in previous studies worldwide. However, most researchers tend to explore the relationship between socio-economic and prevalence of oral diseases. For instance, a systemic review found those in disadvantaged socio-economic status were more susceptible to PD because they had less access to dental care [32]. Several studies have already shown the necessity and social value of covering dental care benefits in healthcare insurance schemes [33–36]. Significantly, our findings provide new insight into this issue as well.

Our synthesis also indicates that when patients get PD, there is usually a process of coping and adaptation, which has already been discussed in many articles [37, 38]. It’s a set of actions aimed at minimizing the adverse impacts of PD. The concrete strategies of coping and adaptation can also be found in other similar articles. Usually, there are two types of coping based on its functions, namely emotion-focused coping and problem-focused coping [39]. In our review, some patients took avoidance actions to maintain their self-esteem and minimize embarrassment. Some, however, tried to solve the problem as thoroughly as possible. Although it’s difficult for some of them to adapt to the disease in a short time, they tried to know more about it and take their own responsibilities seriously, which was beneficial for treatment compliance. Similarly, the findings from a systematic review highlighted the importance of providing information and motivation for adherence to the treatment [40]. Therefore, our results reveal that professionals should identify their coping status and intervene accordingly to help them adapt to the disease better.

Patients living with PD kept self-reflecting and evaluating while getting treatment. When they first learned of their oral condition, they usually actively explored the causes. Different from the findings in quantitative studies [41, 42], some felt that previous dentists’ failure to do their best was a contributing factor to the disease [21, 24]. They felt they were totally under the authority while seeking medical help [24]. Therefore, they have to rely on the present dentists and hope for desired outcomes. The majority of them expressed they had more control over the condition [21, 23–26]. Several quantitative studies have already shown similar findings, in which surgical treatment could improve their OHRQoL [30, 43]. A small number, however, expressed disappointment with the treatment [26], which may be difficult to find in quantitative data. In our review, we found this disappointment may be related to insufficient communication [26]. Therefore, these findings may highlight the importance of effective health education.

In addition, from patients’ perceptions, we can also find that effective and relaxed education from professionals can make a lot of sense to them [25, 26]. As a systematic review has identified, there were various dentist-patient communication skills that can be used to assist in communication curriculum design [44]. A qualitative study conducted in the UK found it was meaningful to engage patients in feedback on dental students’ communication skills in the clinic [45]. Likewise, another qualitative study found that involving patients in medical education could offer a chance for professionals to learn in-depth from patients [46]. Our findings can give a deep insight into patients’ perceptions about the disease and treatment; we hope to provide better dental care in the future.

However, it’s necessary to consider the limitations and quality of the included studies when discussing the findings. In particular, based on the JBI quality appraisal tool, possible biases brought by researchers were not reported in some articles [19, 21, 23–26], which may lower the dependability of the synthesis findings [47]. Moreover, the participants included in this systematic review were from some areas, which may limit the generalizability of findings worldwide. Therefore, more research in this area is needed.

### Limitations and strengths

There are several limitations in the review process. At first, different from our review protocol registered in PROSPERO, we were unable to access Open Grey successfully in the searching process, so we searched Open AIRE instead. Additionally, considering the complexity of different languages and cultures, we only synthesized studies published in English to minimize the possible bias. Therefore, we may have omitted some relevant studies in other languages. Further, our research was limited to qualitative data, so we can’t offer broader insight into patients’ feelings and experiences.

Despite all these limitations, to the best of our knowledge, this meta-synthesis is the first of its kind that provides insight into PD patients’ feelings and experiences of the disease and periodontal treatment. The meta-synthesis was conducted rigorously in various databases, including the grey literature database, to get as many articles as possible. In particular, this review prioritized patients’ voices of living with PD, which should be valued highly in dental practice.

### Implications for clinical practice and future research

Our findings reveal that patient-centered care should be highlighted in PD. Considering various pressure patients experienced, the public should take health education seriously and help them seek medical help earlier to minimize their physical pressure as much as possible. Besides, dental professionals should realize their responsibilities to educate with psychological techniques and, where possible, intervene accordingly to relieve their psychosocial pressure at their best. Policymakers should play a key role in improving the dental health insurance system as far as possible and enabling full coverage of basic screening insurance. Furthermore, these findings from patients’ perspectives, while preliminary, suggest that we can provide more precise and efficient interventions for better compliance and treatment outcomes.

Future research may wish to gather holistic perspectives of patients living with PD in order to improve dental care. Considering the limitations of most included studies, future qualitative research should take measures to minimize potential biases brought by researchers. Additionally, our review only synthesized eight studies, so it’s highly necessary to conduct more research in multiple settings and cultures, including mix-method studies. Further, future studies can focus on understanding the psychological characteristics of PD patients at different stages of the disease based on longitudinal, qualitative studies, which may facilitate designing targeted interventions.

## Conclusion

This meta-synthesis presented a comprehensive understanding of how patients with PD perceived their disease and its treatment. We found these patients generally viewed the disease as a pressure in various aspects, then took coping and adaptation measures towards the condition, and usually engaged in self-reflection and evaluation during the whole process. All these findings could facilitate the provision of patient-centered care in clinical practice.

## Supplementary Information

**Additional file 1**. Details of Searching, Quality Assessment, and Data Analysis.

## Data Availability

PubMed, The Cochrane Library, EMBASE, Scopus, Web of Science, PsycINFO, CINAHL, and Open AIRE.

## Abbreviations

PD: Periodontal disease
PROs: Patient-reported outcomes
OHRQoL: Oral health-related quality of life
JBI: Joanna Briggs institute
