# Supplementary material for: Understanding the feelings and experiences of patients with periodontal disease: a qualitative meta-synthesis
Source: Health Qual Life Outcomes. 2022 Aug 26;20:126. doi: 10.1186/s12955-022-02042-5 (PMC9419312; doi:10.1186/s12955-022-02042-5)
Supplement: Supplementary file 1 — Additional file 1. Details of Searching, Quality Assessment, and Data Analysis. [file 12955_2022_2042_MOESM1_ESM.docx]

**Understanding the feelings and experiences of patients with periodontal disease: a meta-synthesis**

**Supplementary materials**

Appendix. Table S1 Search strategy

Appendix. Table S2 Quality appraisal of the included qualitative studies in JBI

Appendix. Table S3 Examples of data synthesis

**Table S1 Search strategy**

| **Database** | **Indexed and keyword terms** | **N** |
| --- | --- | --- |
| PubMed | #1: (((((("Periodontal Diseases"[MeSH Terms]) OR ("gingival disease*"[MeSH Terms])) OR ("Gingival Recession*"[MeSH Terms])) OR ("Gingivitis"[MeSH Terms])) OR ("Gingival Pocket*"[MeSH Terms])) OR (periodont*[MeSH Terms])) OR (Periodontitis[MeSH Terms]) |  |
|  | #2: ((((experienc*[Title/Abstract]) OR (feeling*[Title/Abstract])) OR (attitude*[Title/Abstract])) OR (perception*[Title/Abstract])) OR (psycho*[Title/Abstract]) |  |
|  | #3: ("mixed study"[Title/Abstract] OR "mixed research"[Title/Abstract]) OR (((("qualitative research"[Title/Abstract]) OR ("qualitative study"[Title/Abstract])) OR ("qualitative methodology"[Title/Abstract])) OR (((((((("phenomenological research"[Title/Abstract]) OR ("grounded theory approach"[Title/Abstract])) OR ("ethnographic research"[Title/Abstract])) OR ("ethnographic study"[Title/Abstract])) OR ("historic research"[Title/Abstract])) OR ("historic study"[Title/Abstract])) OR ("action research"[Title/Abstract])) OR ("case study"[Title/Abstract]))) |  |
|  | #1 AND#2 AND#3 | 39 |
| *Date of Search* | *December 10, 2021 (updated June 21, 2022)* | +2* |
| Cochrane Library | #1 ("periodontal disease*" OR "gingival disease*" OR "gingival recession*" OR gingivitis OR "gingival pocket*" OR periodont* OR periodontitis):ti,ab,kw |  |
|  | #2 (experience* OR feeling* OR attitude* OR perception OR psycho*):ti,ab,kw |  |
|  | #3 (**"qualitative research"** OR **"qualitative study"** OR **"qualitative methodology"** OR **"phenomenological research"** OR **"grounded theORy approach"** OR **"ethnographic research"** OR **"ethnographic study"** OR **"histORic research"** OR **"histORic study"** OR **"action research"** OR **"case study"** OR **"mixed study"** OR **"mixed research"**):ti,ab,kw |  |
|  | #1 AND #2 AND #3 | 4 |
| *Date of Search* | *December 10, 2021 (updated June 21, 2022)* | +0* |
| Scopus | #1：( TITLE-ABS-KEY ( "periodontal disease*" ) OR TITLE-ABS-KEY ( "gingival disease*" ) OR TITLE-ABS-KEY ( "gingival Recession*" ) OR TITLE-ABS-KEY ( "gingivitis" ) OR TITLE-ABS-KEY ( "gingival pocket*" ) OR TITLE-ABS-KEY ( periodont* ) OR TITLE-ABS-KEY ( periodontitis ) ) |  |
|  | #2: ( TITLE-ABS-KEY ( experience* ) OR TITLE-ABS-KEY ( feeling* ) OR TITLE-ABS-KEY ( attitude* ) OR TITLE-ABS-KEY ( psycho* ) OR TITLE-ABS-KEY ( perception ) ) |  |
|  | #3: ( TITLE-ABS-KEY ( "qualitative research" ) OR TITLE-ABS-KEY ( " qualitative study " ) OR TITLE-ABS-KEY ( " qualitative methodology " ) OR TITLE-ABS-KEY ( " phenomenological research " ) OR TITLE-ABS-KEY ( " grounded theory approach " ) OR TITLE-ABS-KEY ( " ethnographic research " ) OR TITLE-ABS-KEY ( " ethnographic study " ) OR TITLE-ABS-KEY ( " historic research " ) OR TITLE-ABS-KEY ( " historic study " ) OR TITLE-ABS-KEY ( " action research " ) OR TITLE-ABS-KEY ( " case study " ) OR TITLE-ABS-KEY ( " mixed study " ) OR TITLE-ABS-KEY ( "mixed research" )) |  |
|  | #1 AND #2 AND #3 | 107 |
| *Date of Search* | *December 10, 2021 (updated June 21, 2022)* | +6* |

(continued on next page)

| **Table S1 (continued)** | | |
| --- | --- | --- |
| **Database** | **Indexed and keyword terms** | **N** |
| Web of Science | #1: TS = ("periodontal disease*" or "gingival disease*" or "gingival recession*" or gingivitis or "gingival pocket*" or periodont* or periodontitis ) |  |
|  | #2: TS= (**experience*** or **feeling*** or **attitude*** or **perception** or **psycho***) |  |
|  | #3: TS= (**"qualitative research"** or **"qualitative study"** or **"qualitative methodology"** or **"phenomenological research"** or **"grounded theory approach"** or **"ethnographic research"** or **"ethnographic study"** or **"historic research"** or **"historic study"** or **"action research"** or **"case study"** or **"mixed study"** or **"mixed research"**) |  |
|  | #1 AND#2 AND#3 | 126 |
| *Date of Search* | *December 10, 2021 (updated June 21, 2022)* | +7* |
| EMBASE (from OVID) | #1: ("periodontal disease*" or "gingival disease*" or "gingival recession*" or gingivitis or "gingival pocket*" or periodont* or periodontitis).mp. [mp=ti, ab, tx, ct, sh, hw, tn, ot, dm, mf, dv, kf, fx, dq] |  |
|  | #2: (experience* or feeling* or attitude* or perception or psycho*).mp. [mp=ti, ab, tx, ct, sh, hw, tn, ot, dm, mf, dv, kf, fx, dq] |  |
|  | #3: **("qualitative research" or "qualitative study" or "qualitative methodology" or "phenomenological research" or "grounded theory approach" or "ethnographic research" or "ethnographic study" or "historic research" or "historic study" or "action research" or "case study" or "mixed study" or "mixed research").mp. [mp=ti, ab, tx, ct, sh, hw, tn, ot, dm, mf, dv, kf, fx, dq]** |  |
|  | #1 AND #2 AND #3 | 224 |
| *Date of Search* | *December 10, 2021 (updated June 21, 2022)* | +17* |
| CINAHL (from EBSCO) | S1: SU "periodontal disease*" OR SU "gingival disease*" OR SU "gingival Recession*" OR SU "gingival pocket*" OR SU periodont* OR SU periodontitis OR SU gingivitis |  |
|  | S2: AB experience* OR AB feeling* OR AB attitude* OR AB psycho* OR AB perception |  |
|  | S3: AB "qualitative research" OR AB "qualitative study" OR AB "qualitative methodology" OR AB "phenomenological research" OR AB "grounded theory approach" OR AB "ethnographic research" OR AB "ethnographic study" OR AB "historic research" OR AB "historic study" OR AB "action research" OR AB "case study" |  |
|  | S4: AB "mixed study" OR AB "mixed research" |  |
|  | S5: S3 OR S4 |  |
|  | S6: S1 AND S2 AND S5 | 9 |
| *Date of Search* | *December 10, 2021 (updated June 21, 2022)* | +1* |

(continued on next page)

**Table S1 (continued)**

| **Database** | **Indexed and keyword terms** | **N** |
| --- | --- | --- |
| PsycINFO | #1: ("periodontal disease*" or "gingival disease*" or "gingival recession*" or gingivitis or "gingival pocket*" or periodont* or periodontitis).mp. [mp=title, abstract, heading word, table of contents, key concepts, original title, tests & measures, mesh word] |  |
|  | #2: (experience* or feeling* or attitude* or perception or psycho*).mp. [mp=title, abstract, heading word, table of contents, key concepts, original title, tests & measures, mesh word] |  |
|  | #3: ("qualitative research" or "qualitative study" or "qualitative methodology" or "phenomenological research" or "grounded theory approach" or "ethnographic research" or "ethnographic study" or "historic research" or "historic study" or "action research" or "case study" or "mixed study" or "mixed research").mp. [mp=title, abstract, heading word, table of contents, key concepts, original title, tests & measures, mesh word] |  |
|  | #1 AND #2 AND #3 | 7 |
| *Date of Search* | *December 10, 2021 (updated June 21, 2022)* | +0* |
| Open AIRE | (periodontal disease* or gingival disease* or gingival recession* or gingivitis or gingival pocket* or periodont* or periodontitis) Result Types: research data; Language: English | 18 |
| *Date of Search* | *December 13, 2021 (updated June 21, 2022)* | +0 |
| Total |  | 567 |

+ N*: number of articles published between December 10, 2021 to June 21, 2022.

**Table S2** **Quality appraisal of the included qualitative studies in JBI**

| **Studies** | **NO.** | **Q1** | **Q2** | **Q3** | **Q4** | **Q5** | **Q6** | **Q7** | **Q8** | **Q9** | **Q10** | **Score** |
| --- | --- | --- | --- | --- | --- | --- | --- | --- | --- | --- | --- | --- |
| Abrahamsson, 2008[1] | #1 | Yes | Yes | Yes | Yes | Yes | No | No | Yes | Yes | Yes | 8 |
| Karlsson, 2009[2] | #2 | Yes | Yes | Yes | Yes | Yes | No | No | Yes | Yes | Yes | 8 |
| Stenman, 2009[3] | #3 | Yes | Yes | Yes | Yes | Yes | No | No | Yes | Yes | Yes | 8 |
| O'Dowd, 2010[4] | #4 | Unclear | Yes | Yes | Yes | Yes | No | No | Yes | Yes | Yes | 7 |
| Horne, 2020 [5] | #5 | Unclear | Yes | Yes | Yes | Yes | Yes | Yes | Yes | Yes | Yes | 9 |
| Pyo, 2020 [6] | #6 | Unclear | Yes | Yes | Yes | Yes | No | No | Yes | Yes | Yes | 7 |
| Hijryana, 2021[7] | #7 | Unclear | Yes | Yes | Yes | Yes | Yes | Yes | Yes | Yes | Yes | 9 |
| Wong, 2021[8] | #8 | Unclear | Yes | Yes | Yes | Yes | No | No | Yes | Yes | Yes | 7 |

Note: “Score” means the number of the answer “yes” out of ten items for each article.

Q1: Is there congruity between the stated philosophical perspective and the research methodology?

Q2: Is there congruity between the research methodology and the research question or objectives?

Q3: Is there congruity between the research methodology and the methods used to collect data?

Q4: Is there congruity between the research methodology and the representation and analysis of data?

Q5: Is there congruity between the research methodology and the interpretation of results?

Q6: Is there a statement locating the researcher culturally or theoretically?

Q7: Is the influence of the researcher on the research, and vice- versa, addressed?

Q8: Are participants, and their voices, adequately represented?

Q9: Is the research ethical according to current criteria or, for recent studies, and is there evidence of ethical approval by an appropriate body?

Q10: Do the conclusions drawn in the research report flow from the analysis, or interpretation, of the data?

**Table S3** **Examples of data synthesis**

| **Analytical themes** | **Descriptive themes** | **Free codes** | **Source** | **Example quotations** |
| --- | --- | --- | --- | --- |
| **Theme 1: Pressure** | Physical | Masticatory discomfort | #4 #5 #7 | If I’m eating a lolly, say if the kids have had one and I’ll eat one, I won’t bite it, I’ll bite it at the back of my teeth #4 (P12) |
|  |  | Poor appearance | #7 | Obviously, my teeth condition disturbs me, especially when I smile or talk. Because of this, I don’t dare to laugh wide open (P16) |
|  |  | Annoying odors | #4 | The wife mentioned something like ‘‘Your breath smells’’ you sort of think ‘‘Ah’’ and you put your hands over your mouth . . . I feel a bit self-conscious (P7) |
|  | Psychosocial | It’s a shock for me | #1 | Well, it’s easy to be shocked ... and of course I wondered about it when she (the dentist) referred me to a specialist, and I hoped it would be ... but the specialist confirmed, instead, that it was periodontitis and told me that I might have had the problem for a long time already, for years, but it was still kind of a shock, you know. |
|  |  | Too much to worry about | #2 #7 | I was at my friend’s house. She asked, “Would you like to eat something?” I replied, “No, I am fine. I currently have loose teeth.” I was so afraid that my teeth would fall out there #7 (P9)  I didn’t feel dizzy, but I felt I could faint because too much blood came out [from her gums] #7 (P4) |
|  |  | Stigma | #1 #4 #5 | “I’ve always felt there was like a stigma about it [periodontal disease]’’ #4 (P13)  “I would love to have a nice smile and maybe whiter teeth, and be proud to smile, as now I just avoid smiling or opening my mouth.” #5 (P2) |

(continued on next page)

| **Table S3 (continued)** | | | | | | | | |  |
| --- | --- | --- | --- | --- | --- | --- | --- | --- | --- |
| **Analytical themes** | | **Descriptive themes** | | **Free codes** | | **Source** | | **Example quotations** | |
|  |  | | Decreased self-confidence | | #7 | | ‘‘It has just affected my self-confidence really and how I feel about myself . . . I was a pretty confident lad you know before that but it has affected my confidence in a big way’’ #4 (P3). | |  |
|  |  |  | Discomfort in communicating with others | | #6 #7 | | I am pretty confident when it comes to everything else. It is just the way my teeth look. I mean, I am thinking about that, when I am talking to people I am thinking ‘‘Are they looking at my teeth’’ #4 (P3) “Since people have been pointed out (my teeth), I automatically cover my mouth with my hands whenever I meet someone. . .some people said harsh things like I look fine with my closed mouth, but I look like a monster when I open my mouth.” #6 (P6)  “Normally, I cover my mouth with my hand when I am aware. I feel shame because I have got lots of missing teeth. I feel different from who I was before. I was confident, but I feel shy now. I can see that I lost so many teeth when I look in the mirror. I think it looks like I only got two teeth when I smile. In the past, I can laugh freely because I still have all of my teeth, but now there is a lack of confidence feeling” #7 (P27) | |  |
|  |  |  | Limitations in daily life | | #1 #2 #6 #7 | | I went through extreme suffering that the meaning of life, I mean I couldn’t find the meaning of it. . .Since I couldn’t chew on it, so I thought about death too.” #6 (P5)  All of my activities were interrupted. I couldn’t do anything. Even sleep felt wrong and still painful. That was awful that I couldn’t do my activities. I couldn’t do anything before the teeth came out by themselves #7 (P16) | |  |
|  | Financial | | Financial cost frustrated me | | #1 #3 | | I feel like my teeth are a part of my body, and my mouth is part of...Well, anyway, I think if you have an illness and have to take medication and all that, I think there ought to be a cost ceiling...Of course I think you should have to pay for part of it yourself, but that there should be subsidised (dental care) like there is for medical care. I really think so. I imagine there are a lot of people, even people who don’t smoke and who live healthy life,who have it (periodontis) ...If you are on two medications that make your mouth dry, then you can get assistance, they say,but in my case there was no help to be had, and I didn’t have the energy to pursue it...it was easier just to pay up. #1 (P1) | |  |
|  |  |  | Economic difficulties | | #6 | | “The cost of expense is too high. I thought it was 2.5 million won as a total, but if the pillar (implant fixture) takes 1 million won, then it would cost me another pretty penny for visiting back and forth.” (P1) | |  |

**References**

1. Abrahamsson KH, Wennström JL, Hallberg U. Patients' views on periodontal disease; attitudes to oral health and expectancy of periodontal treatment: a qualitative interview study. *Oral Health Prev Dent* 2008, 6(3):209-216.

2. Karlsson E, Lymer UB, Hakeberg M. Periodontitis from the patient's perspective, a qualitative study. *International Journal of Dental Hygiene* 2009, 7(1):23-30.

3. Stenman J, Hallberg U, Wennström JL, Abrahamsson KH, Stenman J, Hallberg U, et al. Patients' attitudes towards oral health and experiences of periodontal treatment: a qualitative interview study. *Oral Health & Preventive Dentistry* 2009, 7(4):393-401.

4. O'Dowd LK, Durham J, McCracken GI, Preshaw PM. Patients' experiences of the impact of periodontal disease. *J Clin Periodontol* 2010, 37(4):334-339.

5. Horne PE, Foster Page LA, Leichter JW, Knight ET, Thomson WM. Psychosocial aspects of periodontal disease diagnosis and treatment: A qualitative study. *J Clin Periodontol* 2020, 47(8):941-951.

6. Pyo J, Lee M, Ock M, Lee J. Quality of Life and Health in Patients with Chronic Periodontitis: A Qualitative Study. *Int J Environ Res Public Health* 2020, 17(13):4895.

7. Hijryana M, MacDougall M, Ariani N, Kusdhany LS, Walls AWG. Impact of Periodontal Disease on the Quality of Life of Older People in Indonesia: A Qualitative Study. *JDR Clin Trans Res* 2021:23800844211041911.

8. Wong LB, Kunnasegaran SG, Yap AU, Allen PF. A qualitative study of dental professionals' and patients' knowledge and perceptions of the impact of periodontal disease on systemic health and quality of life. *Community Dent Oral Epidemiol* 2021.
